# Synthesis and biological activity of α-galactosyl ceramide KRN7000 and galactosyl (α1→2) galactosyl ceramide

**DOI:** 10.1016/j.bmcl.2009.05.095

**Published:** 2009-08-01

**Authors:** Natacha Veerapen, Manfred Brigl, Salil Garg, Vincenzo Cerundolo, Liam R. Cox, Michael B. Brenner, Gurdyal S. Besra

**Affiliations:** a School of Biosciences, University of Birmingham, Edgbaston, Birmingham B15 2TT, UK; b Division of Rheumatology, Immunology, and Allergy, Brigham and Women’s Hospital, Harvard Medical School, 1 Jimmy Fund Way, Boston, MA 02115, USA; c Tumor Immunology Group, Weatherall Institute of Molecular Medicine, John Radcliffe Hospital, Headington, Oxford OX1 3QT, UK; d School of Chemistry, University of Birmingham, Edgbaston, Birmingham B15 2TT, UK

**Keywords:** CD1d, KRN7000, *i*NKT, Galactosyl(α1-2)galactosyl

## Abstract

We herein report a faster and less cumbersome synthesis of the biologically attractive, α-galactosyl ceramide (α-GalCer), known as KRN7000, and its analogues. More importantly, the use of a silicon tethered intramolecular glycosylation reaction gave easy access to the diglycosyl ceramide Gal(α1**→**2)GalCer, which has been shown to require uptake and processing to the biologically active α-GalCer derivative.

## Graphical abstract

The synthesis and biological activity of α-galactosyl ceramide (α-GalCer) and Gal(α1→2)GalCer is reported.

CD1d is a nonpolymorphic glycoprotein expressed on the surface of antigen-presenting cells (APCs). It is specifically associated with presenting lipid antigens that activate the distinctive class of T cells, known as invariant Natural Killer T (*i*NKT) cells. *i*NKT cells display characteristics of both T cells and NK cells and play a crucial role in diverse immune responses and other pathologic conditions.^1–4^ When the synthetic glycolipid α-galactosyl ceramide (α-GalCer),^5^ known as KRN7000 (**1**), is bound to CD1d and presented to the T cell receptors (TCRs) on the surface of *i*NKT cells, the latter are activated to release diverse cytokines, including both Th1 and Th2 cytokines.^6–8^ It is believed that the release of Th1 cytokines may contribute to antitumour and antimicrobial functions while that of Th2 cytokines may help alleviate autoimmune diseases^9–11^ such as multiple sclerosis^12^ and arthritis.^13^ α-GalCer and its derivatives have proved to be and remain invaluable tools in understanding the functioning of CD1d and NKT cells in a wide range of immune responses. As part of various ongoing studies, easy access to relatively extensive quantities of these substrates is, therefore, required.

**Figure:**
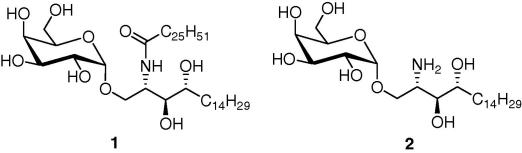

Several elegant syntheses of α-GalCer are present in the literature.^14^ Most of these syntheses make use of benzyl ether protecting groups on the sugar moiety. We, along with many other research groups, have encountered difficulties in the hydrogenolysis of benzyl ethers, thereby leading to low yields of α-GalCer. Therefore, synthetic routes circumventing the problematic hydrogenolysis step are highly desired. Kiso and co-workers^15^ recently reported such a synthesis, where interestingly they also made use of the bulky 4,6-*O*-di-*tert*-butylsilylene (DTBS) group as α-directing in galactosylation donor **3**.

**Figure:**
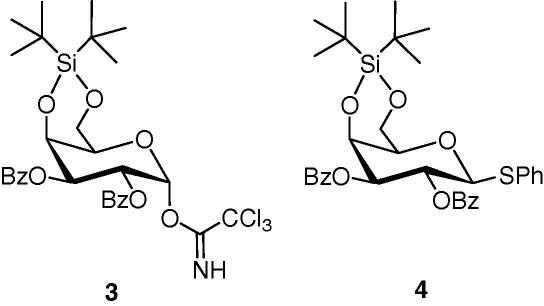

We have employed two different synthetic strategies, including that of Kiso and co-workers to obtain the glycosylated sphingoid base template **2**, which can readily be converted to α-GalCer and other derivatives. Initially, to improve on the reported synthesis we resorted to the use of the thioglycosyl donor **4** as it is easier to handle and has a better shelf life than the trichloroacetimidate donor **3**. Significantly, the second route describes a novel synthesis of α-GalCer and has the additional benefit of providing a crucial intermediate, which can be further modified to yield other biologically active α-GalCer derivatives.

We first synthesized the phytosphingosine acceptor **5**^16^ from (2*S*,3*S*,4*R*)-2-azido-1, 3, 4-octadecanetriol^17^ in three steps as described in Scheme 1.

The thioglycoside **4** was obtained in large scale from commercially available β-d-galactopyranose pentaacetate after standard procedures, as described previously.^18^The critical glycosylation was then attempted on 2 g of the donor **4**. NIS/TfOH activation^18,19^ of thioglycoside **4** (Scheme 2) in anhydrous CH_2_Cl_2_ at −78 °C afforded the glycosylated compound **6** in 71% yield, as the α-anomer exclusively after two hours. Subsequent sequential removal of the silyl group with TBAF and Zemplen’s deprotection of the benzoate protecting groups produced the azide intermediate in quantitative yields after purification by flash chromatography. Different methods for the reduction of the azide group were attempted, including the use hydrogen sulfide, but the best results were obtained via hydrogenation in methanol. 800 mg of the amine **2** was hence isolated as a white solid.

Scheme 3 illustrates our novel strategy to synthesise α-GalCer and its derivatives. Silicon tethered intramolecular glycosylation^20^ is a particularly attractive method for generating glycosidic bonds stereoselectively, but not attempted by many research groups due to the difficulty in handling and relative instability of the silylene-tethered sugar derivative. However, successful examples have been reported and we were motivated by Bols^21^ work on the synthesis of disaccharides containing α-galactosyl linkages. Hence, 3, 4, 6-tri-*O*-acetylgalactopyranosyl chloride (**7**)^22^ was obtained from β-d-galactopyranose pentaacetate and converted to the corresponding thioglycoside **8** by reacting with thiophenol in the presence of caesium carbonate. The tethered compound **9** was then synthesised following the procedure described by Bols.^21^ Rearrangement of the silylene **9**, catalyzed by *N*-iodosuccinimide (NIS) in anhydrous nitromethane at 80 °C,^21^ yielded the desired product **10** along with some small amounts of **5** after 2 h. It was observed that by careful monitoring and quenching of the reaction as soon as compound **9** was consumed, helped in minimizing the regeneration of phytosphingosine derivative **5** and hence enhanced the yield of the glycosylated product. Methanolysis, followed by hydrogenation of the azide then afforded compound **2**. Finally, N-acylation with the fully saturated fatty acid, hexacosanoic acid, was achieved via reaction of the corresponding acid chloride with the free amine **2** in a 1:1 mixture of THF and saturated sodium acetate solution. Target compound **1** was obtained as a white solid after concentration of the organic phase and purification of the residue by flash chromatography. The spectroscopic data of the latter were consistent with the literature.^14^

While our first approach (Scheme 2) is more direct and higher yielding, the alternative route (Scheme 3) also provides the additional benefit of freeing the hydroxyl group at C-2 on the sugar residue (compound **10)**. This allows for selective modification on α-GalCer; such as the introduction of an additional sugar residue. Specifically, the diglycosyl ceramide, Gal(α1**→**2GalCer) **12** has been used to study lysosomal glycolipid processing.^23^ Briefly, galactosidases from lysosomes are responsible for truncating oligoglycosyl ceramides to monoglycosyl ceramides before they can bind to CD1d and be presented to *i*NKT cells.^23^ Scheme 4 depicts a new strategy for synthesizing disaccharide **11** via NIS/TfOH activation of sugar donor **4** at −78 °C in anhydrous CH_2_Cl_2_ for 3 h. Once more, the directing effect of the bulky silyl group ensured the formation of the desired α-linkage, as confirmed by the H-1 and C-1 signals in ^1^H and ^13^C NMR. Compound **12** was obtained after routine procedures, similar to those described above and exhibited spectroscopic data consistent with the literature.^24^

We next tested the biological activity of α-GalCer and Gal(α1**→**2GalCer). Both lipids stimulated human and mouse *i*NKT cells in the presence of CD1d-expressing antigen-presenting cells (APC) (Fig. 1a and b). α-GalCer, but not Gal(α1**→**2GalCer), stimulated *i*NKT cells in an APC-free CD1d-Fc fusion protein plate assay (Fig. 1c). In fix/pulse, pulse/fix experiments α-GalCer stimulated an *i*NKT cell response under both conditions, whereas Gal(α1**→**2GalCer) resulted in cytokine release only under the pulse/fix condition (Fig. 1d). Together, these data suggest that α-GalCer and Gal(α1**→**2GalCer) described here can stimulate human and mouse *i*NKT cells. Furthermore, Gal(α1**→**2GalCer), in contrast to α-GalCer, required uptake and processing to generate the biologically active monoglycosyl ceramide.

In conclusion, we have demonstrated the versatility of both compounds **4** and **10** as crucial intermediates in practical and high-yielding syntheses of α-GalCer and other biologically important derivative, such as Gal(α1**→**2GalCer).^26^

## Figures and Tables

**Figure 1:**
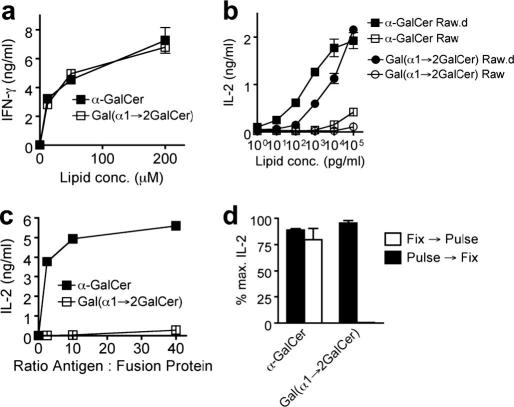
α-GalCer and Gal(α1**→**2GalCer) stimulate CD1d-restricted *i*NKT cells. (a) In vitro activation of 2.5 × 10^4^ human *i*NKT cells (clone BM2a.3) in co-culture with 2.5 × 10^4^ U937 cells and various concentrations of α-GalCer (filled squares) and Gal(α1**→**2GalCer) (open squares). After 16 h, cytokines were determined in culture supernatants by ELISA; (b) in vitro activation of 5 × 10^4^ mouse *i*NKT cells (hybridoma DN32) in co-culture with 5 × 10^4^ RAW cells transfected with CD1d (filled symbols) or untransfected (open symbols) and various concentrations of α-GalCer (squares) and Gal(α1**→**2GalCer) (circles). After 16 h, cytokine concentrations were determined in culture supernatants by ELISA; (c) Plate-bound murine recombinant CD1d-Fc fusion proteins were loaded with α-GalCer (filled squares) or Gal(α1**→**2GalCer) (open squares) for 16 h, washed, and 5 × 10^4^*i*NKT cell hybridomas were added per well. Cytokines were determined in culture supernatants by ELISA; (d) RAW cells transfected with CD1d were pulsed with 100 ng/ml of α-GalCer or Gal(α1**→**2GalCer) for 3 h and then washed and fixed with glutaraldehyde (filled bars), or fixed and then pulsed for 3 h (open bars). 10^5^ APCs were co-cultured with 10^5^*i*NKT cell hybridomas for 16 h and cytokines were determined in culture supernatants by ELISA. Cytokine responses are expressed as percent of maximal response. Methods are described elsewhere.^25^

**Scheme 1:**
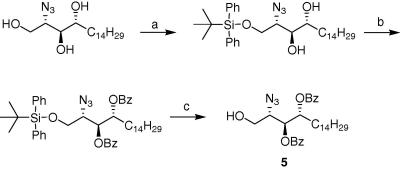
Reagents and conditions: (a) TBDPSCl, Pyr, quant; (b) BzCl, Pyr, 88%; (c) TBAF, THF, 82%.

**Scheme 2:**
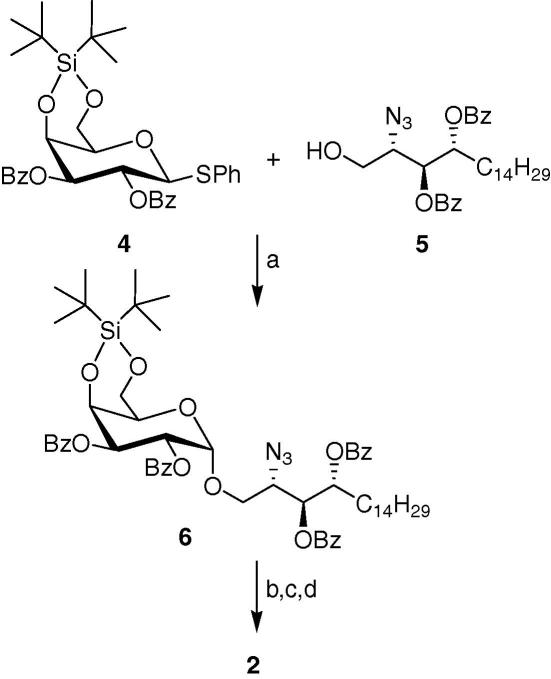
Reagents and conditions: (a) NIS/TfOH, CH_2_Cl_2_, 67%; (b) TBAF, THF, quant; (c) NaOMe/MeOH, 92%; (d) H_2_, Pd, 88%.

**Scheme 3:**
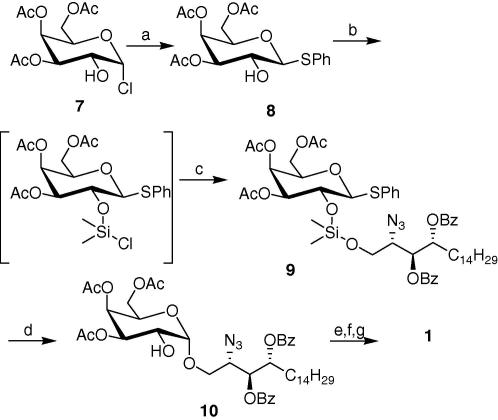
Reagents and conditions: (a) PhSH, DMF, Cs_2_CO_3_, 75%; (b) (CH_3_)_2_Si(Cl)_2_, Pyridine, CH_2_Cl_2_, quant; (c) **5**, Pyridine, DMF, 72%; (d) NIS, CH_3_NO_2_, 66%; (e) NaOMe/MeOH, quant; (f) H_2_, Pd/C, MeOH, 80%; (g) C_25_H_51_COCl, THF, NaOAc, 75%.

**Scheme 4:**
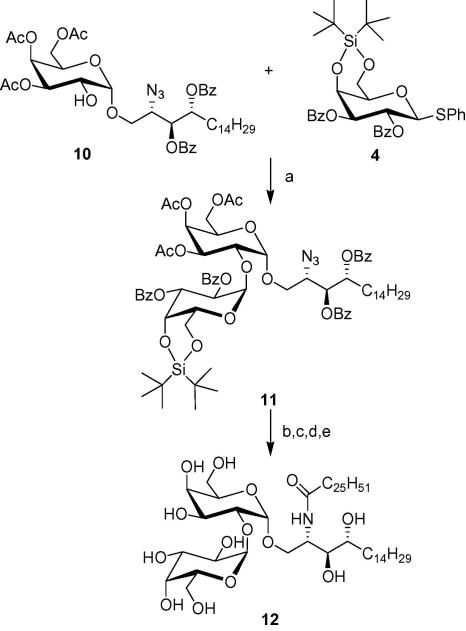
Reagents and conditions: (a) NIS/TfOH, CH_2_Cl_2_, 67%; (b) TBAF, THF, quant; (c) NaOMe/MeOH, quant; (d) H_2_, Pd, MeOH, 80%; (e) C_25_H_51_COCl, THF, NaOAc, 78%.
